# Supplementary material for: Effect of Thrombin on the Metabolism and Function of Murine Macrophages
Source: Cells. 2022 May 23;11(10):1718. doi: 10.3390/cells11101718 (PMC9139186; doi:10.3390/cells11101718)
Supplement: Supplementary file 1 [file cells-11-01718-s001.zip › supp.pdf]

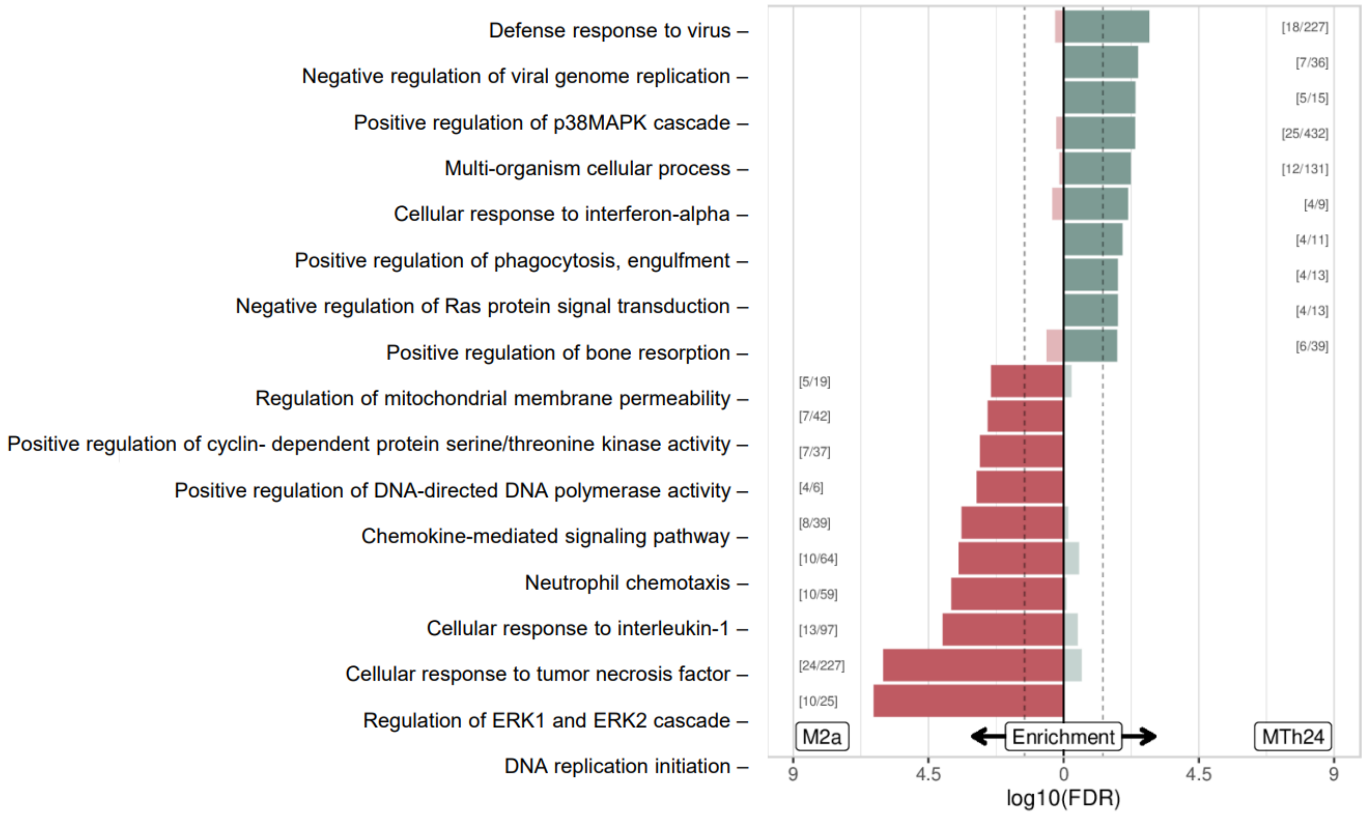

Figure S1. **Thrombin induced changes in macrophage gene expression.** Gene set enrichment analysis for top 10 differentially regulated gene ontology (GO) terms relating to the comparison of murine bone marrow monocyte-derived macrophages polarized to the M2a phenotype or incubated with thrombin (0.1 U/mL, 24 hours).
